# Imaging for local recurrence of breast cancer

**DOI:** 10.1007/s00432-024-05709-2

**Published:** 2024-04-17

**Authors:** T. Schlaiss, L. Bott, S.-L. Herbert, C. Bartmann, M. Kiesel, J. Salmen, S. T. Sauer, S. A. Christner, B. Petritsch, J.-P. Grunz, A. Woeckel, S. Löb, J. Diessner

**Affiliations:** 1 Department of Gynaecology and Obstetrics, University Hospital Würzburg, Josef-Schneider-Str. 4, 97080 Würzburg, Germany; 2 Department of Diagnostic and Interventional Radiology, University Hospital Würzburg, Josef-Schneider-Str. 4, Würzburg, Germany; 3 Department of Diagnostic and Interventional Radiology, Klinikum Klagenfurt Am Wörthersee, Klagenfurt Am Wörthersee, Austria

**Keywords:** Imaging methods, Hereditary breast cancer, Isolated locoregional recurrence, Contralateral breast cancer

## Abstract

**Purpose:**

Isolated locoregional recurrence of breast cancer (ILRR) and contralateral breast cancer (CBC) affect up to 20% of all breast cancer (BC) patients in the first 20 years after primary diagnosis. Treatment options comprise surgical interventions and further systemic therapies depending on the histological subtype. Patients with hereditary breast or ovarian cancer (HBOC) undergo MRI, mammography, and ultrasound in the aftercare of BC, while non-HBOC (nHBOC) patients do not regularly receive MRI. Since early detection is crucial for morbidity and mortality, the evaluation and constant improvement of imaging methods of the breast is necessary.

**Methods:**

We retrospectively analyzed the data of 1499 former BC patients that received imaging of the breast at a tertiary-care university hospital between 2015 and 2020. The analysis comprised various patient characteristics, such as breast density, age, tumor size and subtype, and their influence on BC detection rates by the different imaging methods.

**Results:**

Within the patient sample, 176 individuals (11.7% of former BC patients) were diagnosed with either ILRR or CBC. CBC was observed in 32.4% of patients, while both ILRR and secondary breast cancer occurred in 20.5% and 23.9% of all patients. Sensitivity of MRI, mammography, and ultrasound for recurrent malignancy was 97.9%, 66.3%, and 67.8%, respectively. ILRR and CBC detection rates were similar for patients with and without HBOC history. Lower breast density and larger tumor size increased the detection rates of all imaging modalities.

**Conclusion:**

In breast cancer survivors, MRI might improve the early detection of ILRR and CBC in both HBOC and nHBOC patients.

## Introduction

Isolated locoregional recurrence of breast cancer (ILRR) can affect the breast, chest wall or axillary lymph nodes. It occurs in 2 to 20% during the first 20 years after primary disease (Pan et al. 2017). The likelihood for ILRR increases with aggressive histological subtypes, such as triple-negative breast cancer (TNBC) or advanced primary tumors including positive lymph nodes or a lack of complete remission after neoadjuvant chemotherapy. Due to advances in systemic therapies for BC, rates for ILRR decreased over time. Several studies using targeted therapeutics showed improved disease-free or event-free survival when used for primary breast cancer (Gianni et al. 2016; Masuda et al. 2017; Schmid et al. 2022; Tutt et al. 2021; von Minckwitz et al. 2019). Contralateral breast cancer (CBC) occurs in 10.2% within the first 20 years after primary disease and is also depending on initial cancer treatment (Xiong 2018).

While therapeutical concepts of breast cancer were increasingly individualized during the last decades, aftercare programs to identify recurrent disease such as ILRR and CBC are equal for patients with all subtypes of BC. During the first 3 years after primary diagnosis, patients should receive clinical examinations four times a year. For the 4th and 5th year after diagnosis, they should receive clinical examination two times a year. Six years after primary diagnosis, patients are examined once a year. Imaging of the breast is planned annually using mammography and ultrasound. Patients with hereditary breast cancer (HBOC – hereditary breast and ovarian cancer) and documented mutation in e.g., BRCA1/2 genes have increased risk for CBC. Cumulative risks by time since first BC are up to 60% for BRCA1 mutation carriers and 68% for BRCA2 mutation carriers (Kuchenbaecker et al. 2017). Thus, breast imaging and early detection of ILRR and CBC play a major role. Even a risk-reducing surgery of the contralateral breast should be considered and discussed in dependency of the prognosis of the primary disease.

For patients with hereditary background, such as familial cancer history, increased calculated risk for pathogen mutation or detected mutations, an intensified-aftercare program has been established in various countries. In Germany, such patients receive annual MRI, mammography and ultrasound (Bick et al. 2019). Depending on the mutation detected, another interval ultrasound can be recommended.

Early detection of recurrent disease is crucial. In case of early detection, survival rates vary from 65% in case of ILRR after breast-conserving therapy and radiation to 50% in case of ILRR of the chest wall after mastectomy (Haffty et al. 1991). Detection rates for ILRR and CBC are described to be around 59–99% depending on the imaging modality (Lee et al. 2021).

Breast composition is defined as the density of fibroglandular breast tissue and is categorized as a (entirely fatty), b (scattered areas of fibroglandular density), c (heterogeneously dense), and d (extremely dense) according to the American College of Radiology (ACR) (ACR 2019). Breast density is an independent risk factor for the development of BC (Malkov et al. 2016).

Treatment options for ILRR comprise wide excisions in case of a chest wall affection or mastectomy in case of ILRR after breast-conserving therapy. Repeated breast-conserving therapy for ILRR after breast-conserving therapy and radiotherapy can be discussed with patients on an individual basis. Veronesi et al. found that further local tumor reappearance was 15.2% of patients with tumors of < 2 cm size and occurence > 48 months after initial treatment. In contrast, patients with ILRR of > 2 cm tumor size and occurence < 48 months after initial treatment presented with further local tumor reappearance in 31.2% (Gentilini et al. 2012). Surgical interventions of the axillary lymph nodes after lymph node resection for primary disease are indicated only in case of clinically affected lymph nodes.

Systemic treatment for ILRR is recommended in case of HER2 + and/or triple-negative subtypes. Based on the CALOR trial, patients with hormone receptor negative ILRR benefitted from chemotherapy. Seventy % of those patients were breast cancer free after 10 years, while only 34% of those without chemotherapy were. Moreover, they also showed better overall survival (Wapnir et al. 2018). In case of inoperability due to tumor size or location of ILRR, systemic treatment is recommended to be used according to the treatment algorithms of metastasized situations (Gluz/Heil 2022). Treatment of CBC is recommended according to treatment strategies for primary breast cancer depending on the tumor subtype.

In summary, early detection and treatment of ILRR and CBC is highly important for the prognosis of patients. In this analysis, we investigated a subgroup of patients with ILRR and CBC that were treated at our institution with the focus on the detection rates by different imaging modalities.

## Methods

### Study design and inclusion criteria

We retrospectively analyzed all patients with prior breast cancer diagnosis that received diagnostic procedures or treatments at a tertiary-care university hospital between 2015 and 2020. Ethical approval was unnecessary due to the retrospective design of the study. The need for additional written informed consent was waived by the local ethics committee (reference number 2163183).

Inclusion criteria comprised prior diagnosis of invasive breast cancer or ductal carcinoma in situ (DCIS), actual diagnosis of recurrent disease or secondary carcinoma of the breast (ILRR or CBC), and existence of at least two examinations using different imaging methods (MRI, ultrasound or mammography). We also included patients with recently diagnosed carcinoma in situ, while patients with ipsilateral or contralateral recurrent disease in axillary lymph nodes were excluded from the analysis.

Data were extracted from the hospitals electronic data system (SAP, Walldorf, Germany), and the picture archiving and communication system (Merlin, Phönix-PACS, Freiburg, Germany). Imaging studies were analyzed according to the Breast Imaging and Reporting Data System (BI-RADS) [ACR, BI-RADS Atlas 5th Edition] Standardized double reading for mammography was used. Ultrasound examinations were mainly performed by subspecialized gynecologists.

In case of unambiguous radiological reports, the images were re-evaluated by two board-certified radiologists with at least 7 years of experience in senology based on consensus reading as part of this study.

### Patient data

We assessed patients ‘ characteristics such as age at the time of diagnosed ILRR/CBC, sex, BI-RADS classification, BMI, HBOC history, breast density based on ACR, tumor pathology/subtype, and initial treatment for BC (e.g., breast-conserving therapy, mastectomy, sentinel node biopsy, axillary dissection, chemotherapy, anti-HER2-directed therapy and endocrine therapy).

### Statistical analysis

Frequencies and percentages of patients with ILRR and CBC were assessed. We described characteristics of HBOC and nHBOC subgroups and the aftercare imaging that was performed for each subgroup.

For statistical analysis of differences between the HBOC and the nHBOC group, we used the Chi-square test and Fisher`s exact test. P-values of p < 0.05 were considered indicative of statistical significance.

Binary logistic regression was calculated for the detection rates of mammography and ultrasound. The detection rate was the nominally-scaled dependent variable and modeled dichotomously (0 = “false-negative”, 1 = “true-positive”). Independent variables were HBOC, tumor size (= pT status), breast density, presence of DCIS, and age.

Statistical analyses were performed using SPSS (IBM SPSS Statistics Version 27).

## Results

We identified 1499 patients with a history of BC that received imaging of the breast between 2015 and 2020. 213 (14.2%) patients showed ILRR or CBC.We further analyzed 176 patients (11.7%) that were diagnosed with ILRR or CBC and met the inclusion criteria. Within the final study sample, 174 patients (98.9%) were female and 2 (1.2%) were male. In 43 cases (24.4%), patients received aftercare in the national program of families with HBOC. The median time to relapse was 129 months (range 6 to 674). Time to ILRR after breast-conserving therapy was 136 months (range 12 to 674) and 85 months (range 15 to 368) after mastectomy.The median time to CBC was 120 months (range 6 to 511).

CBC was diagnosed in 32.4% of individuals. In 20.5% ipsilateral recurrent breast cancer and in 23.9% ipsilateral secondary breast cancer (divergent histological subtype in comparison to primary disease) were diagnosed. ILRR and CBC detection rates did not differ significantly for patients with and without HBOC history (p = 0.478). Patients with HBOC history were significantly younger, pre-/perimenopausal, and of normal BMI when compared to the nHBOC group. Breast cancer of no special type (NST) was the most common histopathological subtype (63.6%), while invasive lobular cancer was detected in 11.4% of all cases. Invasive lobular cancer affected nHBOC patients in 14.3%, while HBOC patients showed invasive lobular cancer in 2.3% (p = 0.25). Tumor size smaller than 2 cm (T1) or DCIS was diagnosed in 75.4% of all patients. The most frequently diagnosed subtype was Luminal A (hormone receptor positive, Ki67 low) (48.3%), while TNBC was diagnosed in 22.2%. When divided in groups of HBOC or nHBOC, an association with molecular subtypes was ascertained (p = 0.030). 39.5% of patients presenting with TNBC had HBOC status, while 16.5% were part of the nHBOC subgroup. Chemotherapy was applicated in 28 (65.1%) HBOC and 38 (28.6%) nHBOC patients, endocrine therapy was applicated in 17 (39.5%) HBOC and 63 (47.4%) nHBOC patients. Anti-HER2 directed therapy was applicated in 7 of 23 (30.4%) HER2 positive patients. Further patient characteristics are displayed in Table 1.

**Table 1:** Basic characteristics of the nHBOC and HBOC patient samples

Variable	nHBOC	HBOC	p-value
Age (years)			
≤ 49	9 (6.8%)	21 (48.8%)	< *0.001*^*#*^
≥ 50	124 (93.2%)	22 (51.2%)	
Menopausal status			
Pre-/perimenopausal	15 (11.3%)	21 (48.8%)	< *0.001*^*#*^
Postmenopausal	116 (87.2%)	22 (51.2%)	
Missing values	*2 (1.5%)*		
BMI			
≤ 24.9	48 (36.1%)	26 (60.5%)	< *0.001*^*#*^
≥ 25	85 (63.9%)	13 (30.2%)	
Missing values		*4 (9.3%)*	
Breast density			
A	20 (15%)	4 (9.3%)	0.057^*^
B	70 (52.6%)	16 (37.2%)	
C	36 (27.1%)	18 (41.9%)	
D	4 (3%)	4 (9.3%)	
Missing values	*3 (2.3%)*	*1 (2.3%)*	
Secondary tumors and recurrences			
Ipsilateral secondary tumor	35 (26.3%)	7 (16.3%)	0.478^*^
Ipsilateral recurrence	25 (18.8%)	11 (25.6%)	
Contralateral secondary tumor	43 (32.3%)	14 (32.6%)	
Contralateral recurrence	5 (3.8%)	2 (4.6%)	
Ipsilateral DCIS	13 (9.8%)	2 (4.6%)	
Contralateral DCIS	11 (8.3%)	7 (16.3%)	
Contralateral LCIS	1 (0.7%)	0 (0%)	
Histological types of breast cancer			
NST	83 (62.4%)	29 (67.4%)	0.250^*^
Invasive lobular carcinoma	19 (14.3%)	1 (2.3%)	
DCIS	24 (18%)	10 (23.2%)	
LCIS	1 (0.8%)	0 (0%)	
Others	6 (4.5%)	3 (7%)	
Molecular subtypes of breast cancer			
Luminal A	70 (52.6%)	15 (34.9%)	0.030^*^
Luminal B (HER2 -)	24 (18%)	5 (11.6%)	
Luminal B (HER2+)	11 (8.4%)	4 (9.3%)	
HER 2+	6 (4.5%)	2 (4.7%)	
TNBC	22 (16.5%)	17 (39.5%)	
pT status			
pTis	22 (16.5%)	8 (18.6%)	0.603^*^
pT1mic-1	75 (56.4%)	28 (65.1%)	
pT2a-c	27 (20.3%)	4 (9.3%)	
pT3a-c	3 (2.3%)	1 (2.3%)	
pT4a-d	6 (4.5%)	2 (4.7%)	
pN status			
pN0	113 (85%)	39 (90.6%)	0.126^*^
pN1	16 (12%)	2 (4.7%)	
pN2	3 (2.3%)	0 (0%)	
pN3	1 (0.8%)	2 (4.7%)	
pM status			
pM0	125 (94%)	42 (97.7%)	*0.307*^*#*^
pM1	8 (6%)	1 (2.3%)	
Grading			
G1	10 (7.5%)	1 (2.3%)	0.167^*^
G2	78 (58.6%)	20 (46.5%)	
G3	35 (26.4%)	16 (37.2%)	
Missing values	*10 (7.5%)*	*6 (14%)*	
Ki67			
< 25	90 (67.7%)	23 (53.5%)	*0.067*^*#*^
≥ 25	43 (32.3%)	20 (46.5%)	

P-value < 0.05 is considered statistically significant

^#^Statistical significance has been tested by Fisher’s exact test

^*^Statistical significance has been tested by Chi-square test

For diagnosing ILRR or CBC, 160 patients (90.1%) received mammography, 174 patients (98.9%) received ultrasound, and 96 (54.5%) received MRI of the breast.

**Fig. 1:**
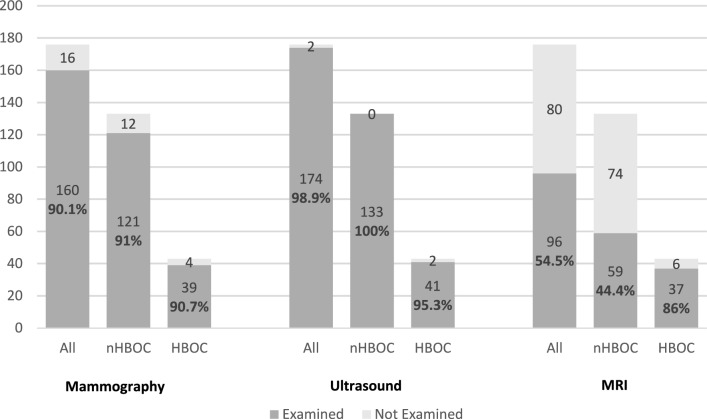
Breast imaging modality. The absolute and relative number of patients receiving mammography, ultrasound and MRI for breast imaging are displayed. MRI is more often performed in the HBOC collective

HBOC patients received mammography in 90.7% (39/43), ultrasound in 95.3% (41/43), and MRI in 86% (37/43). Patients of the standard aftercare program received mammography in 91%, ultrasound in 100%, and MRI in 44.4% (Fig. 1).

In Table 2, we show the distribution of patients receiving all three or only two imaging methods for the diagnosis of ILRR/CBC. In 78 patients, all three imaging modalities were performed and for 29 (37.2%) of those, the diagnosis was possible in all modalities. In 12 patients (15.4%), ILRR/CBC was diagnosed based on ultrasound and MRI only and in 11 (14.1%) cases on mammography and MRI only. In 26 cases (33.3%), recurrent malignancy was diagnosed only in MRI. In 80 cases of ILRR/CBC, two imaging modalities (ultrasound and mammography) were performed. In 50 cases (62.5%), the correct diagnosis was determined in both modalities while 16 cases (20%) were detected in ultrasound only and 14 (17.5%) using mammography only.

**Table 2:** Distribution of patients receiving three or two imaging modalities

Use of modalities	nHBOC	HBOC	all
All modalities used	47 (60.3%)	31 (39.7%)	78
Correctly diagnosed by all	20 (69%)	9 (31%)	29
Only detected by US + MRI	8 (66.7%)	4 (33.3%)	12
Only detected by MX + MRI	4 (36.4%)	7 (63.6%)	11
Only US + MX used	74 (92.5%)	6 (7.5%)	80
Correctly diagnosed by all	45 (90%)	5 (10%)	50
Only detected by US	16 (100%)	0 (0%)	16
Only detected by MX	13 (92.9%)	1 (7.1%)	14

Mammography (Mx), ultrasound (US) and magnetic resonance imaging (MRI) of the breast

Sensitivity was 66.3% for mammography, 67.8% for ultrasound, and 97.9% for MRI examinations (Table 3).

**Table 3:** Sensitivity of each breast imaging modality for ILRR and CBC

	Incorrectly diagnosed (BIRADS 1–3)	Correctly diagnosed (BIRADS 4–6)
Mammography		
All	54 (33.8%)	106 (66.3%)
nHBOC	39 (32.2%)	82 (67.8%)
HBOC	15 (38.5%)	24 (61.5%)
*p*-value	0.299	
Ultrasound		
All	56 (32.2%)	118 (67.8%)
nHBOC	35 (26.3%)	98 (73.7%)
HBOC	21 (51.2%)	20 (48.8%)
*p*-value	0.003	
MRI		
All	2 (2.1%)	94 (97.9%)
nHBOC	1 (1.7%)	58 (98.3%)
HBOC	1 (2.7%)	36 (97.3%)
*p*-value	0.625	

P-value < 0.05 is considered statistically significant

The binomial logistic regression model of mammography was statistically significant (p = 0.017), resulting in a small amount of explained variance, as shown by Nagelkerke’s R^2^ = 0.115. Overall percentage of accuracy in classification was 70.3%, with a sensitivity of 89.4% and a specificity of 33.3%.

The binomial logistic regression model of ultrasound was statistically significant (p =  < 0.001), also resulting in a small amount of explained variance, as indicated by Nagelkerke’s R^2^ = 0.207. Classification was 72.8%, with a sensitivity of 84.3% and a specificity of 48.1%. Patients with higher breast density were less likely to receive a correct ILRR or CBC diagnosis by conventional imaging techniques only (p < 0.05). We found that in 77.8% of patients with ACR c/d, ILRR was diagnosed using mammography and ultrasound, whereas 88.6% of patients with breast density ACR a/b were diagnosed correctly using conventional imaging. When using ultrasound, we found an association with tumor size (p = 0.021) and existence of DCIS components (p = 0.004). Tumors ≥ 2 cm were 3.4 times more likely diagnosed by sonography and tumors in the absence of DCIS four times more likely. (Table 4a and b). Detection of ILRR or CBC using mammography was associated with breast density and tumor size. Lower breast density and bigger tumor size increased detection rates. Patients with breast density a/b were twice as frequently diagnosed and those with tumor size ≥ 2 cm 3.2 times more frequently (Table 5a and b).

**Table 4:** Variables associated with the correct diagnosis of ILRR or CBC in ultrasound

A binominal logistic regression model was calculated using the variables HBOC, pT status, breast density, DCIS, and age. Categories, encoding and frequencies of these variables are displayed in (a)			
Variables	Categories	Encoding	Frequency
HBOC	HBOC	0	39
	nHBOC	1	130
pT status	Tis + pT1	0	128
	≥ pT2	1	41
Breast density	A + B	0	109
	C + D	1	60
DCIS	DCIS	0	33
	no DCIS	1	136
Age	≤ 49 years	0	27
	≥ 50 years	1	142

The overall accuracy was 72.8% with a sensitivity of 84.3% and a specificity of 48.1% (b)							
	S.E	Wald	df	Sig	OR	Upper	Lower
HBOC	0.473	5.571	1	0.018	3.055	1.209	7.725
pT status	0.528	5.287	1	0.021	3.366	1.196	9.470
Breast density	0.384	0.296	1	0.587	1.232	0.581	2.615
DCIS	0.432	8.481	1	0.004	3.519	1.509	8.207
Age	0.547	0.033	1	0.856	1.105	0.378	3.225
Constant	0.625	5.260	1	0.022	0.238		

The logistic regression yielded a p-value of < 0.001. P-values < 0.05 were considered statistically significant

Displays signficance (Sig.), odds ratio (OR) and confidence interval (upper and lower) of each variable

**Table 5:** Variables associated with the correct diagnosis of ILRR or CBC in mammography

A binominal logistic regression model was calculated using the variables HBOC, pT status, breast density, DCIS, and age. Categories, encoding and frequencies of these variables are displayed in (a)			
Variables	Categories	Encoding	Frequency
HBOC	HBOC	0	38
	nHBOC	1	120
pT status	Tis + pT1	0	117
	≥ pT2	1	41
Breast densitiy	A + B	0	100
	C + D	1	58
DCIS	DCIS	0	33
	no DCIS	1	125
Age	≤ 49 years	0	26
	≥ 50 years	1	132

The overall accuracy was 70.3% with a sensitivity of 89.4% and a specificity of 33.3% (b)							
	S.E	Wald	df	Sig	OR	Upper	Lower
HBOC	0.484	0.016	1	0.899	0.941	0.364	2.428
pT status	0.458	6.454	1	0.011	3.199	1.304	7.847
Breast density	0.368	4.852	1	0.028	0.445	0.216	0.915
DCIS	0.461	3.126	1	0.077	0.443	0.180	1.092
Age	0.549	0.329	1	0.566	1.370	0.467	4.015
Constant	0.613	3.507	1	0.061	3.150		

The logistic regression yielded a p-value of < 0.03. P-values < 0.05 were considered statistically significant

Displays signficance (Sig.), odds ratio (OR) and confidence interval (upper and lower) of each variable

## Discussion

The early detection of ILRR and CBC plays a major role for morbidity and mortality of BC survivors (Dunst et al. 2001). The knowledge about risks is highly imported in counseling patients. There is evidence about the increased risk for CBC for BRCA1 or 2 mutation carriers depending on their age at primary diagnosis and the time since primary diagnosis (Kuchenbaecker et al. 2017). Thus, HBOC BC survivors can already take their CBC risks into account when planning primary surgery including prophylactic procedures of the contralateral breast or remain in an intensified imaging program.

While diagnostic modalities have improved significantly over time and there are lots of new potent-targeted therapies available, one should scrutinize our restrained approach of the aftercare management of all BC patients that is based on the motto “one fits all”. Even though we already have evidence that MRI is improving detection rates, we still perform mammography and ultrasound screening only in the aftercare of BC in case of the absence of HBOC history (Eisen et al. 2024).The use of breast MRI is generally restricted to specific situations such as HBOC history or CUP (cancer of unknown primary) syndrome. Moreover, starting in 2001, national health insurances recognize the use of MRI as helpful for the diagnosis of ILRR after breast-conserving therapy or after mastectomy with reconstruction of the breast using implants. Since then, they financially support breast MRI in case of uncertain findings of mammography and ultrasound in patients with BC history (G-BA 2001). However, a routine MRI for the detection of ILRR or CBC is still not considered the standard of care.

Since, the majority of literature concerning ILRR/CBC detection is several decades old, we further analyzed latest data in our breast cancer center over a 5-year period of time. In our institution, we found a rate for ILRR or CBC of 12.6% which is congruent with the literature (Pan et al. 2017; Xiong 2018). Of those patients, 23.8% had a history of HBOC, which also reflects common knowledge.

Detection of ILRR and CBC in rT1 stages was achieved in 73% of all patients. It is known that outcome depends on early detection, since there is scientific evidence that tumor size is a risk factor for another ILRR (re-recurrence) (Wapnir et al. 2006). Furthermore, we were able to show that those patients that did not receive MRI were more often diagnosed with higher tumor size (T stage). In most cases, BC of NST was diagnosed, which is known to be the most common histological subtype (Strehl et al. 2011). But we also found that invasive lobular BC was mainly detected in nHBOC patients (14.1%), while HBOC patients were diagnosed with invasive lobular BC in only 2.2%. Of note, while CDH1 mutations are associated with invasive lobular carcinoma, those mutations are found in less than 1% of all BC patients (Euhus 2014). In contrast, BRCA1 germline pathogenic variants are not associated with an invasive lobular subtype (Yadav et al. 2021). Based on our analyses, we also found that HBOC patients more often showed aggressive tumor subtypes with Ki67 > 25%.

Higher breast density was associated with younger age and led to decreased detection of ILRR and CBC in mammography (Yeom et al. 2019). The percentage of patients with ILRR and CBC was equally high in HBOC patients and patients without a history of HBOC. This fact is interesting since one would expect a higher rate of CBC in patients with HBOC. It has been shown previously that especially those HBOC patients with pathogenic mutations for BRCA1 and 2 have an increased risk for CBC depending on the age at initial diagnosis (Kuchenbaecker et al. 2017). One reason might be the low amount of patientis in this group. Another reason that there is still a relevant amount of patients with familial cancer history suggestive of HBOC who have not been counseled or tested for pathogenic mutations. But since, we discuss risk-reducing surgery on the contralateral breast for those patients with pathogenic variants of BRCA1/2 based on the risk of about 30% to develop CBC, the presented data is of crucial importance.

HBOC patients who received an intensified-aftercare program showed more often ILRR with the same histological subtype, while those patients without a history of HBOC more often showed a divergent histological subtype for ILRR. There is no literature available about this fact. We assume that the reason lies within the tumorigenic potential/behavior of the breast tissue in HBOC patients, while there might have been multifocal or multicentric primary disease with undetected divergent histopathology in nHBOC patients or ILRR might be based on incomplete primary resection status. It is known that pathogenic variants of BRCA1 are associated with triple-negative subtypes and those of BRCA2 with hormone receptor positive subtypes (Engel et al. 2020).

We detected the highest sensitivity for the detection of ILRR or CBC using MRI. Lee et al. analyzed the use of MRI in the aftercare of BC patients after breast-conserving therapy. They found that detectability for recurrent disease was significantly higher using MRI (99%) in comparison to mammography (59.4%) and ultrasound (68.9%). They further identified early fast enhancement as a major feature to detect recurrent disease after surgery of the breast (Lee et al. 2021). These data are in line with our findings and underline the importance of MRI.

CBC after risk-reducing surgical treatments in HBOC patients needs to be further investigated. There are publications showing CBC rates of up to 11% despite of bilateral mastectomy (Allue Cabanuz et al. 2020). On the contrary, Van Sprundel et al. showed a significantly reduced risk for CBC after risk-reducing surgery (1.3% vs. 46.4%) and a 5-year OS of 94% vs. 77% for BRCA1/2 mutation carrier (van Sprundel et al. 2005). Influencing factors might be the applied surgical technique and thus the amount of removed breast tissue. Breast MRI might help to identify postoperative remaining breast tissue. But there is still no evidence about assessment criteria of relevant remaining tissue in MRI.

Evidence for HBOC patients with moderate penetrance gene mutations is even less available. Also taken into consideration are analyses on patient reported outcomes. There are statements that the fear of cancer recurrence was higher in patientin with contralateral risk-reducing procedures (Srethbhakdi et al. 2020).

There are several attempts to improve and individualize the aftercare of patients with BC according of their risks and needs. One of those is the follow up program called BETTER-CARE (“BrEasT cancer aftTERCARE follow up and program “), a German study that investigates a multidisciplinary approach to improve patients’ quality of life. Funded by the German federation, it also comprises digital solutions for improved networking of all professionals involved in the aftercare of a patient (Wöckel and Heuschmann 2024).

Limitations of this study are the retrospective design and a relatively low amount of patients included in the analysis. There are several aspects, e.g., economical, false-positive and false-negative results of an imaging modality, as well as side effects that could not be assessed due to the study design but that play major role in identifying an optimized aftercare. In search of an optimized aftercare, one should also take into account that the majority of literature available is old and reflects times before individualized therapy concepts became the standard of care.

## Conclusion

We provide evidence that the use of MRI is more sensitive in the detection of ILRR and CBC in both HBOC and nHBOC patients. Therefore, one should consider modifying the current aftercare programs for BC survivors. In light of a relevant number of individualized and targeted therapy options that positively affect a patient’s prognosis, one should take individual patient characteristics into account when designing an optimized diagnostic workup in BC aftercare. Further prospective studies are highly needed.

## Data Availability

The data sets generated and analyzed during the current study are available from the corresponding author on reasonable request.
